# IMGT/StatClonotype for Pairwise Evaluation and Visualization of NGS IG and TR IMGT Clonotype (AA) Diversity or Expression from IMGT/HighV-QUEST

**DOI:** 10.3389/fimmu.2016.00339

**Published:** 2016-09-09

**Authors:** Safa Aouinti, Véronique Giudicelli, Patrice Duroux, Dhafer Malouche, Sofia Kossida, Marie-Paule Lefranc

**Affiliations:** ^1^IMGT^®^, The international ImMunoGeneTics information system^®^, Laboratoire d’ImmunoGénétique Moléculaire LIGM, Institut de Génétique Humaine IGH, UPR CNRS 1142, Montpellier University, Montpellier, France; ^2^Higher School of Statistics and Information Analysis, Unité Modélisation et Analyse Statistique et Economique, University of Carthage, Tunis, Tunisia

**Keywords:** IMGT/HighV-QUEST, IMGT-ONTOLOGY, immunoglobulin, antibody, T cell receptor, next generation sequencing, immunoinformatics, statistical significance

## Abstract

There is a huge need for standardized analysis and statistical procedures in order to compare the complex immune repertoires of antigen receptors immunoglobulins (IG) and T cell receptors (TR) obtained by next generation sequencing (NGS). NGS technologies generate millions of nucleotide sequences and have led to the development of new tools. The IMGT/HighV-QUEST, available since 2010, is the first global web portal for the analysis of IG and TR high throughput sequences. IMGT/HighV-QUEST provides standardized outputs for the characterization of the “IMGT clonotype (AA)” (AA for amino acids) and their comparison in up to one million sequences. Standardized statistical procedures for “IMGT clonotype (AA)” diversity or expression comparisons have recently been described, however, no tool was yet available. IMGT/StatClonotype, a new IMGT^®^ tool, evaluates and visualizes statistical significance of pairwise comparisons of IMGT clonotype (AA) diversity or expression, per V (variable), D (diversity), and J (joining) gene of a given IG or TR group, from NGS IMGT/HighV-QUEST statistical output. IMGT/StatClonotype tool is incorporated in the R package “IMGTStatClonotype,” with a user-friendly interface. IMGT/StatClonotype is downloadable at IMGT^®^^1^ for users to evaluate pairwise comparison of IG and TR NGS statistical output from IMGT/HighV-QUEST and to visualize, on their web browser, the statistical significance of IMGT clonotype (AA) diversity or expression, per gene, the comparative analysis of CDR-IMGT and the V–D–J associations, in immunoprofiles from normal or pathological immune responses.

## 1 Introduction

The adaptive immune responses of humans and other jawed vertebrate species (gnathostomata) are characterized by the B and T cells and the extreme diversity of their respective antigen receptors, the immunoglobulins (IG) or antibodies and the T cell receptors (TR) (up to 2.10^12^ different IG and TR specificities per individual) (1, 2). IMGT^®^, the international ImMunoGeneTics information system^®^^2^ (3), created in 1989 by Marie-Paule Lefranc (Montpellier University and CNRS) to manage the huge and complex diversity of these antigen receptors, is at the origin of immunoinformatics, a science at the interface between immunogenetics and bioinformatics (4).

Next generation sequencing (NGS) generates millions of IG and TR nucleotide sequences allowing analysis of the adaptive immune repertoires. IMGT/HighV-QUEST is the first web portal for the NGS analysis of IG and TR (5, 6), based on IMGT-ONTOLOGY (7) and freely available at IMGT^®^. IMGT/HighV-QUEST provides a standardized output, including the characterization of the “IMGT clonotype (AA)” (AA for amino acids) diversity or expression (8), and their comparison in up to one million sequences. “IMGT clonotype (AA)” is defined as a unique V–(D)–J rearrangement (IMGT genes and alleles determined at the nucleotide level), conserved CDR3-IMGT anchors (cysteine C 104, tryptophan W 118 or phenylalanine F 118), and a unique CDR3-IMGT AA junction sequence. IMGT clonotype (AA) diversity is the number of IMGT clonotypes (AA) per V, D, or J gene, and the IMGT clonotype (AA) expression is the number of sequences assigned, unambiguously, to a given IMGT clonotype (AA) per V, D, or J gene (9).

IMGT^®^ has recently defined a standardized procedure for evaluating the statistical significance of pairwise comparisons between differences in proportions of the IMGT clonotype (AA) diversity or expression, per gene of a given IG or TR group (9), from IMGT/HighV-QUEST statistical output. To make available the results issued from this standardized procedure and for a comparative analysis of CDR-IMGT and V-D-J associations, IMGT^®^ developed a new tool, IMGT/StatClonotype, incorporated in the R package “IMGTStatClonotype,” with a user-friendly interface. IMGT/StatClonotype performs pairwise comparison of IG and TR NGS results, from the IMGT/HighV-QUEST statistical output. IMGT/StatClonotype is described here for the first time, using as an example, a set of B cell NGS sequences. IMGT/StatClonotype is downloadable at http://www.imgt.org/StatClonotype/.

## 2 Design and Implementation

### 2.1 IMGT/StatClonotype tool

IMGT/StatClonotype is an IMGT^®^ tool, incorporated in the R (10) package “IMGTStatClonotype,” which performs evaluation and visualization of pairwise comparisons of IMGT clonotype diversity or expression, per V (variable), D (diversity), and J (joining) gene of a given IG or TR group, from NGS IMGT/HighV-QUEST statistical output, through a user-friendly web interface implemented using Shiny framework (11) in users’ own browser. Comparative analysis is performed per IMGT gene and, for the first time, per IMGT allele (for genes with significant differences). Additional functionalities include analysis of CDR-IMGT length and CDR-IMGT AA properties per IMGT AA classes and heatmaps of V-D-J associations.

### 2.2 “IMGTStatClonotype” R package

The “IMGTStatClonotype” R package is downloadable at IMGT^®^, at the IMGT/StatClonotype web page, see text footnote 1. The installation mode is fully described in the IMGT/StatClonotype Documentation. The package manual, the “IMGTStatClonotype” source package for users familiarized with R and example sets for testing the tool are also available at the IMGT/StatClonotype web page.

### 2.3 IMGT/StatClonotype Interface

**Figure 1:**
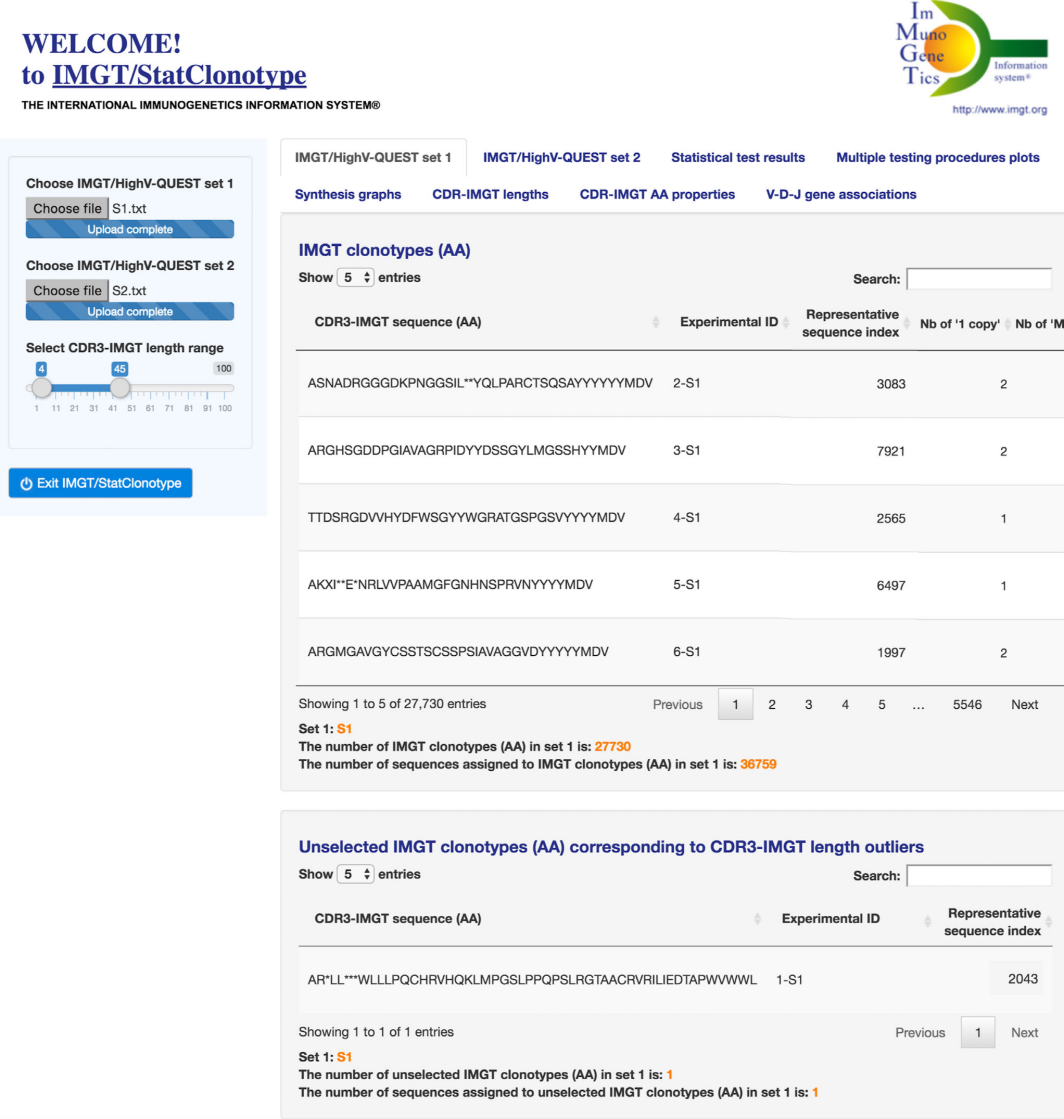
**IMGT/StatClonotype interface with its two panels**. In the left panel, users choose the files of the two sets to be compared and can select the CDR3-IMGT length range of IMGT clonotypes (AA) (by default CDR3-IMGT lengths ≥4 AA and ≤45). In the right panel, users can choose among eight tabs at the top: “IMGT/HighV-QUEST set 1,” “IMGT/HighV-QUEST set 2,” “Statistical test results,” “Multiple testing procedures plots,” “Synthesis graphs,” “CDR-IMGT lengths,” “CDR-IMGT AA properties,” and “V–D–J gene associations.” The display shown here is for “IMGT/HighV-QUEST set 1.”

The IMGT/StatClonotype interface comprises two panels. In the left panel (Figure 1), users choose the files of the two IMGT/HighV-QUEST sets to be compared. These files correspond, for each set, to the file “stats_xxx” from the data directory of the IMGT/HighV-QUEST statistical output and have to be previously uploaded by the user. Users can select the CDR3-IMGT length range of IMGT clonotypes (AA) to be analyzed (by default CDR3-IMGT lengths ≥4 AA and ≤45). CDR3-IMGT length outliers are eliminated from the statistical procedure.

**Figure 2:**
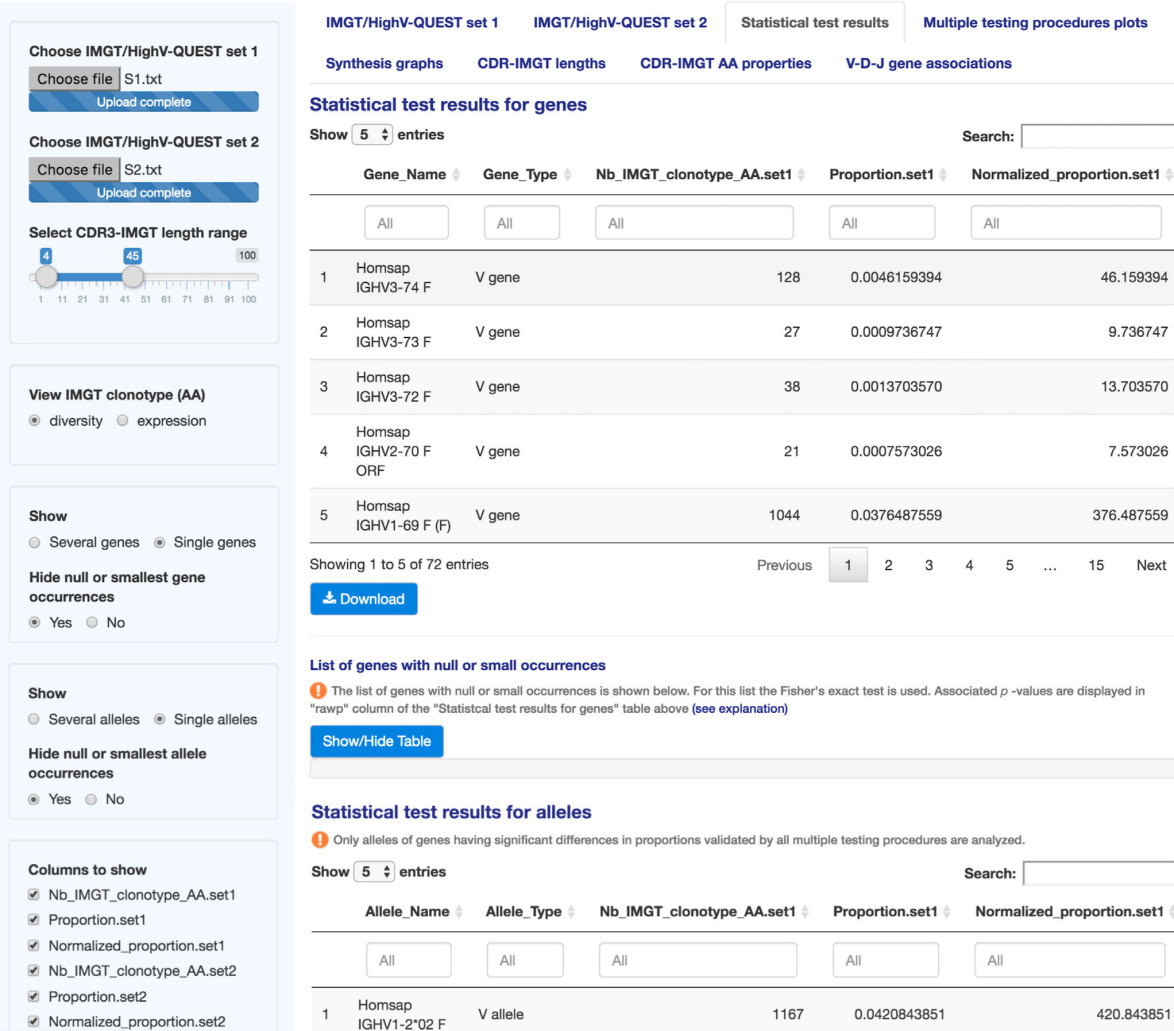
**IMGT/StatClonotype Statistical test results**. Statistical test results are displayed for genes (upper part) and for alleles (lower part). The results are displayed in 21 columns made visible by scrolling horizontally and downloadable in csv files. The content of the columns is described in Table S2 in Supplementary Material.

**Figure 3:**
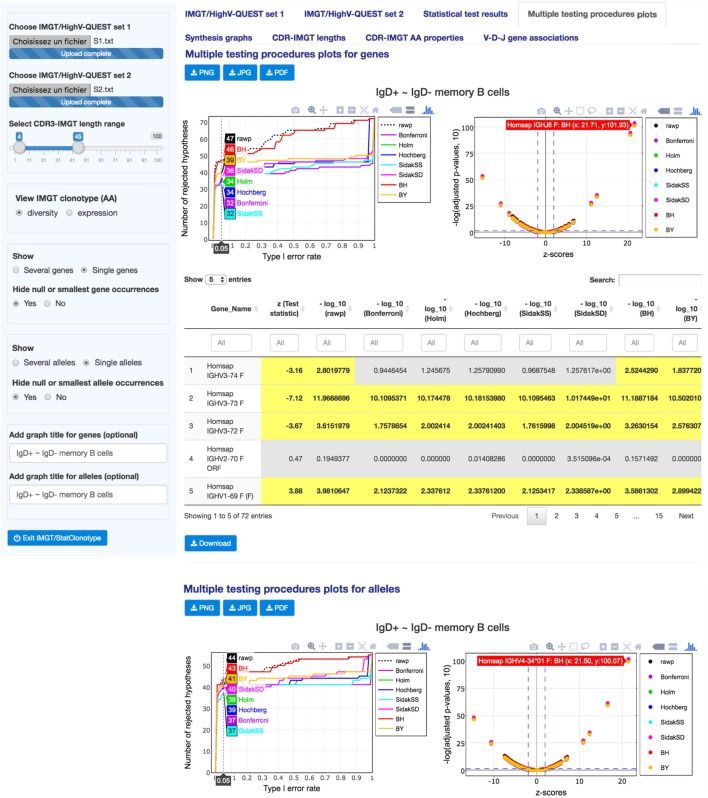
**IMGT/StatClonotype Multiple testing procedures plots**. “Multiple testing procedures plots” displays interactive plots for genes (upper part) and for alleles (lower part), which comprise line graphs (left figures) and scatter plots (right figures). Values of the scatter plots [negative decimal logarithms (−log_10_) of unadjusted *p*-values (black symbols) and adjusted *p*-values obtained by each multiple testing procedure (colored symbols) and *z*-scores] are reported in a table below the figures (downloadable in csv files). All graphs are downloadable in three image formats: PNG, JPG, and PDF.

**Figure 4:**
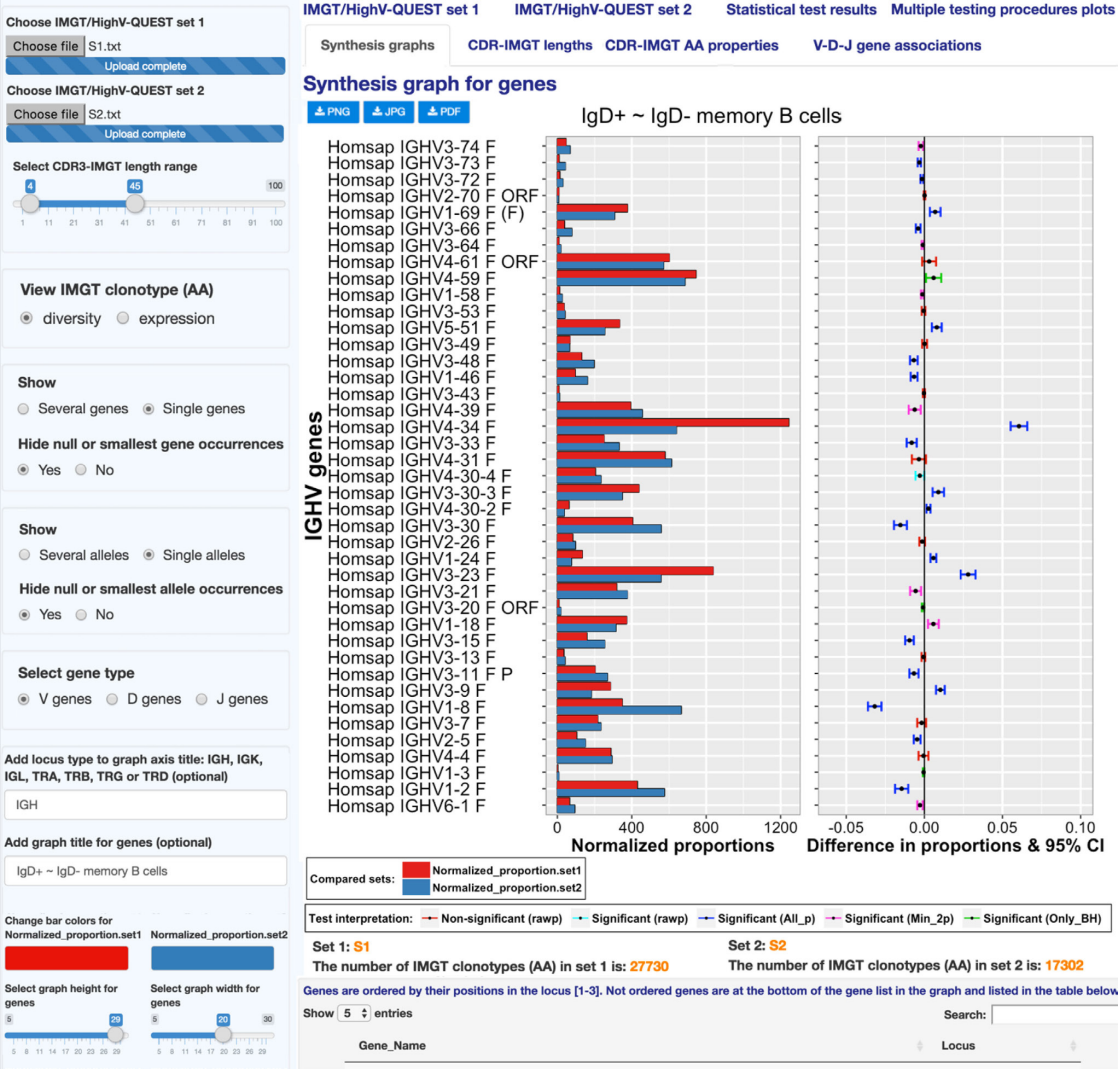

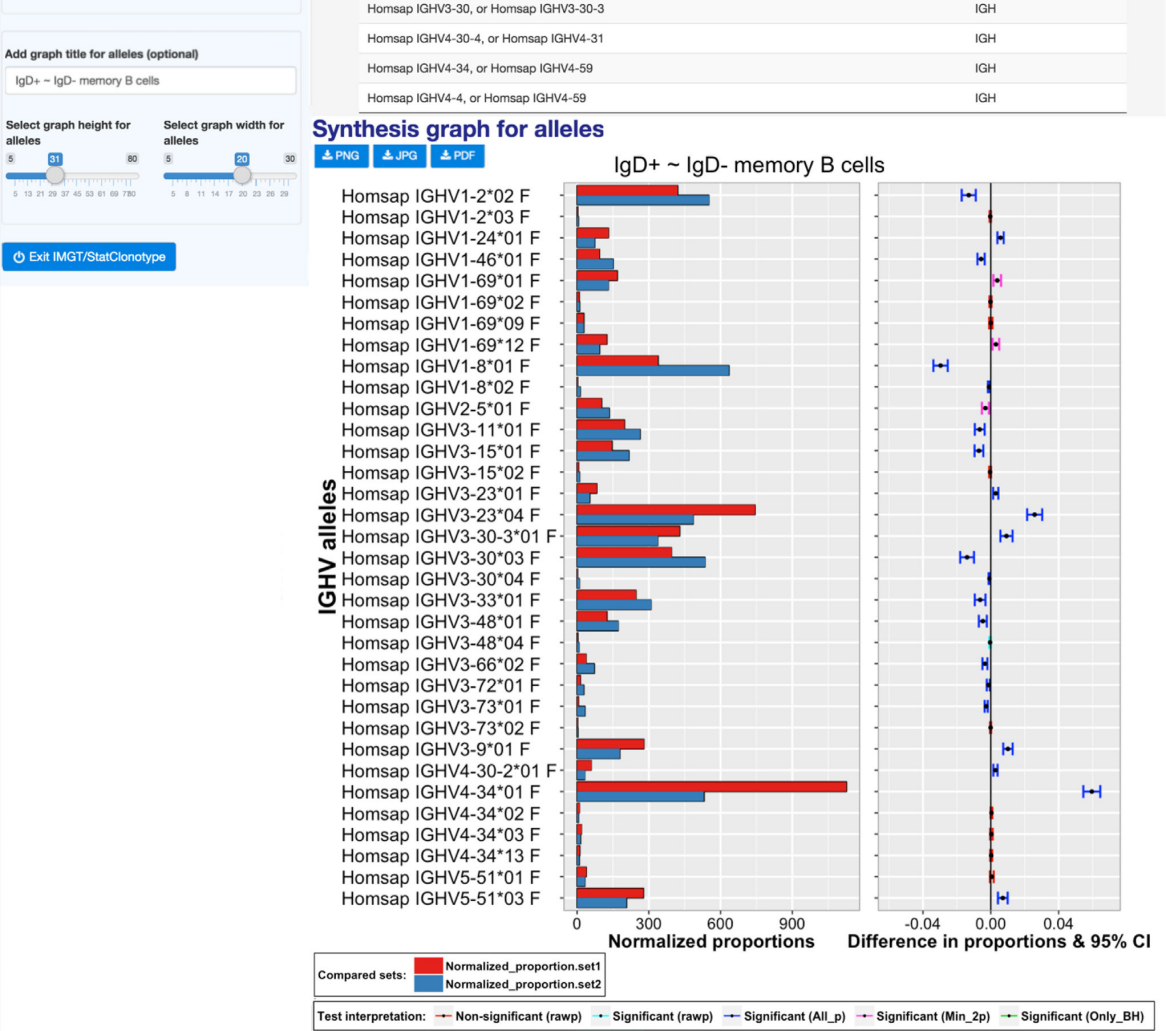
**IMGT/StatClonotype Synthesis graphs**. Synthesis graphs are displayed for genes (upper part) and for alleles (lower part). Synthesis graphs combine a normalized bar graph of gene or allele proportions and the differences in proportions with significance and confidence intervals (CI). Synthesis graphs are downloadable in different formats (PNG, JPG, and PDF). The height and the width of the graph can be adjusted to improve the clarity of the axes text.

**Figure 5:**
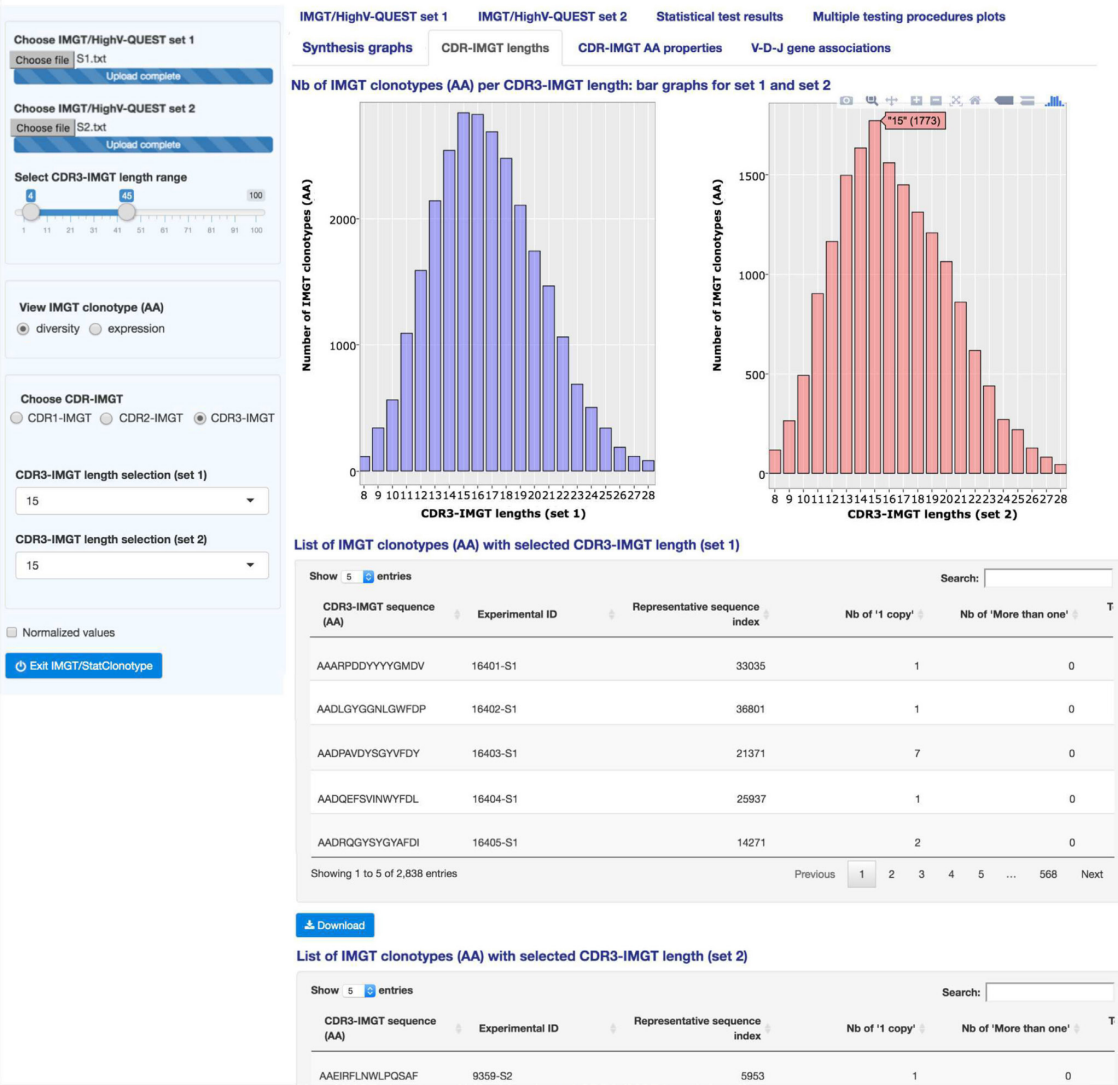
**IMGT/StatClonotype CDR-IMGT lengths**. “CDR-IMGT lengths” displays interactive bar graphs for the distribution of IMGT clonotypes (AA) (for IMGT clonotype diversity) or distribution of sequences assigned to IMGT clonotypes (AA) (for IMGT clonotype expression) for a given CDR-IMGT type. Bar graphs are interactive showing, for a given CDR-IMGT length, the number of IMGT clonotypes (AA) or number of sequences between brackets. The lists of CDR3-IMGT sequences of the IMGT clonotypes (AA), for a selected CDR3-IMGT length, are given per set in tables downloadable in csv files. Bar graphs are downloadable in PNG format.

**Figure 6:**
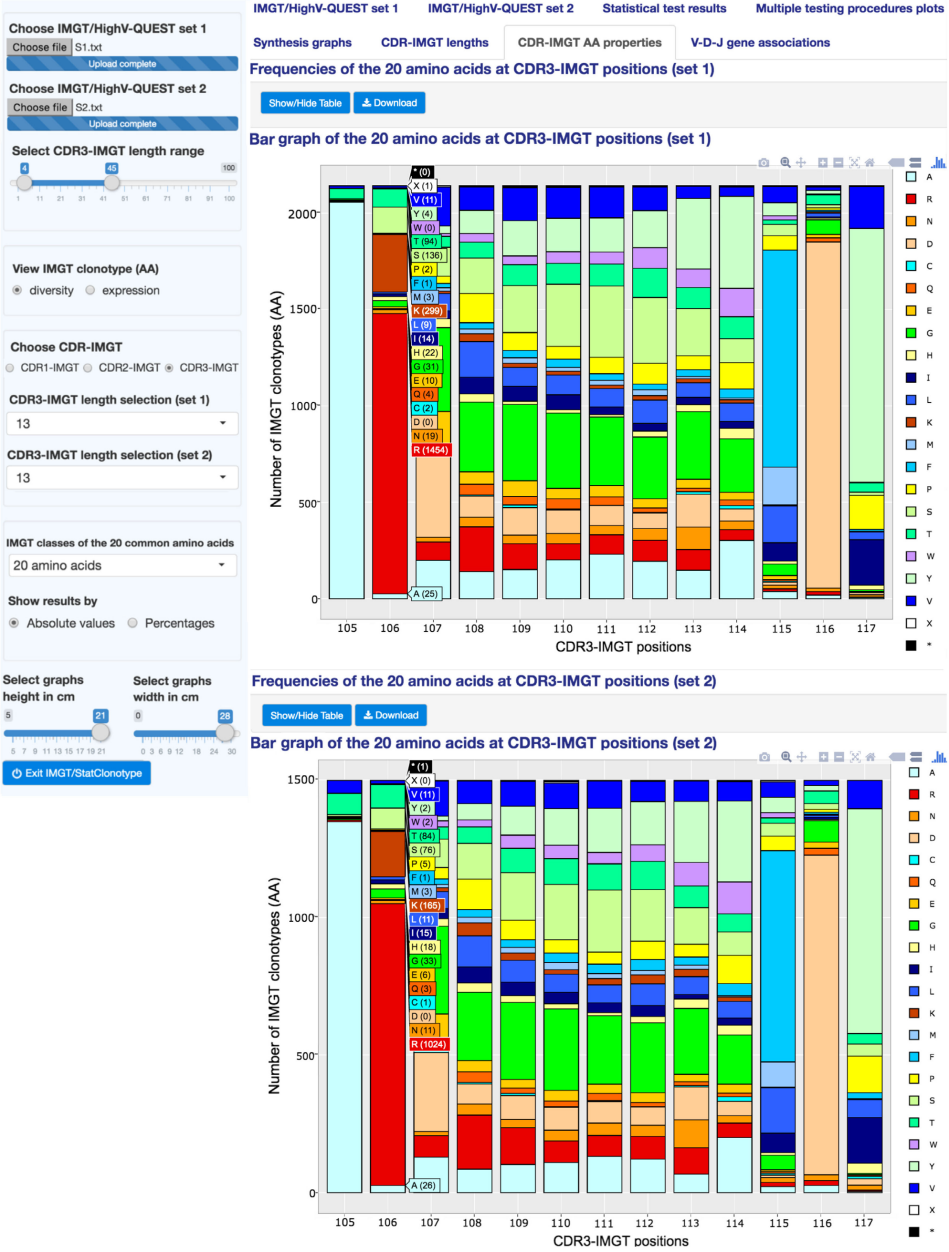

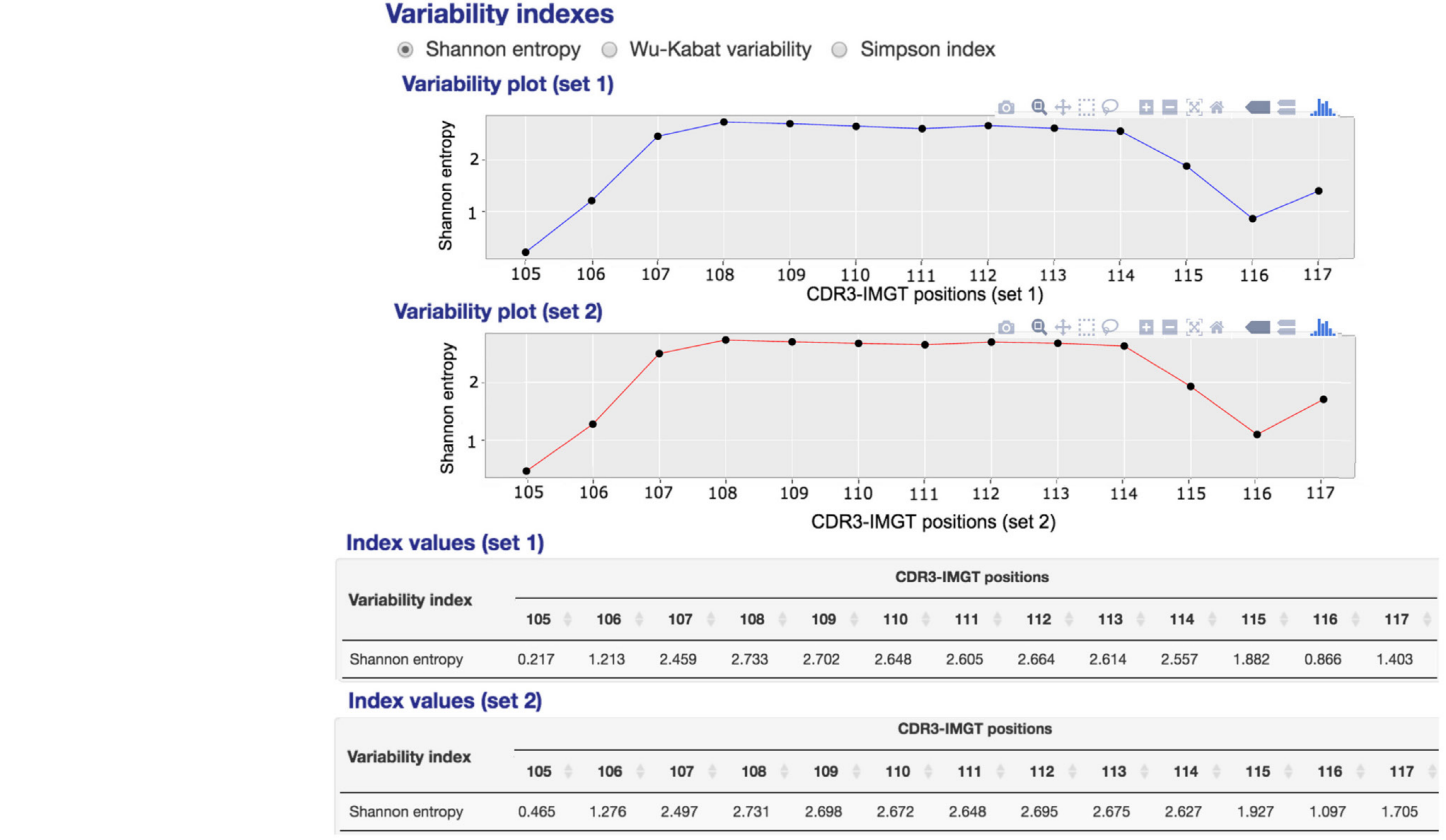
**IMGT/StatClonotype CDR-IMGT AA properties**. “CDR-IMGT AA properties” displays the distribution of the 20 common amino acids (or of the IMGT AA class representatives) at a specific CDR-IMGT position. Variability plots based on “Shannon entropy,” “Wu-Kabat variability,” and “Simpson index” indexes are generated. To visualize AA (or IMGT AA class representatives) frequencies at a specific CDR-IMGT position, interactive stacked bar graphs are generated for each set. IMGT classes of the 20 common amino acids are shown under the mouse cursor with corresponding frequencies shown between brackets in absolute or percentage values. Stacked bar graphs are downloadable in PNG format.

**Figure 7:**
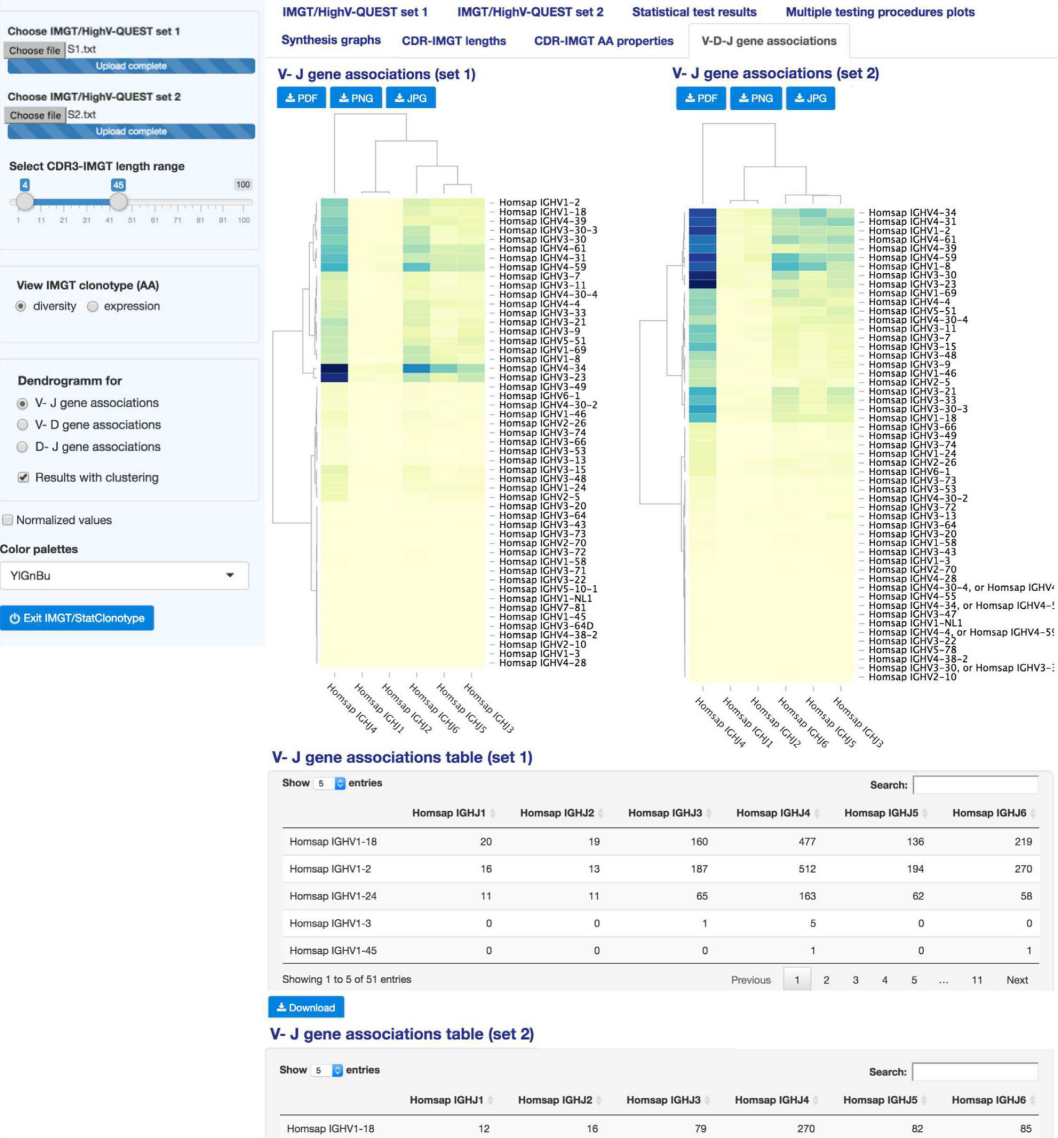
**IMGT/StatClonotype V–D–J gene associations**. “V–D–J gene associations” displays heatmaps to represent V–J, V–D, or D–J gene associations in sets 1 and 2. Heatmaps are downloadable in different formats (PNG, JPG, and PDF). Under heatmaps, tables crossing the V–J, V–D, or D–J gene occurrences in sets 1 and 2 are given and downloadable in csv files. The clustered heatmaps of the V–J associations in sets 1 and 2 are shown as examples.

In the right panel (Figure 1), users can choose among eight tabs at the top: “IMGT/HighV-QUEST set 1,” “IMGT/HighV-QUEST set 2,” “Statistical test results,” “Multiple testing procedures plots,” “Synthesis graphs,” “CDR-IMGT lengths,” “CDR-IMGT AA properties,” and “V-D-J gene associations.” The display shown is for “IMGT/HighV-QUEST set 1.” A similar display is obtained for “IMGT/HighV-QUEST set 2.” The six other tabs correspond to table and graph results displays, described in the section “Results” (Figures 2–7). For each display, users can select corresponding parameters in the left panel.

### 2.4 Data Sets

Two sets, out of six sets from Ref. (12), were chosen as examples for illustrating the features offered by IMGT/StatClonotype. Sets “S1” and “S2” correspond, respectively, to 43,558 reads of IgD^+^ and 28,142 reads of IgD^−^ memory B cells isolated from a healthy female subject, obtained using the Roche GLFLX 454 technology. Sequencing data are available in the NCBI Sequence Read Archive under the accession code SRP037774 (“S1”: SRX470417 and “S2”: SRX470416). The reads were analyzed with IMGT/HighV-QUEST program version 1.1.3, IMGT/V-QUEST program version 3.2.31 and IMGT/V-QUEST reference directory release 201338-1. Resulting IMGT/HighV-QUEST “stats_xxx” files (S1.txt and S2.txt) are available as data sets examples for IMGT/StatClonotype at http://www.imgt.org/StatClonotype/.

## 3 Results

### 3.1 IMGT/HighV-QUEST Sets 1 and 2

“IMGT/HighV-QUEST set 1” and “IMGT/HighV-QUEST set 2” provide for each set to be compared a table of the IMGT clonotypes (AA). Only those within the selected CDR3-IMGT length range (left panel) are displayed and compared (Figure 1). The number of IMGT clonotypes (AA) and the number of sequences assigned to IMGT clonotypes (AA) are given below the table. For example, for S1, with a default CDR3-IMGT length range (4–45 AA), the number of IMGT clonotypes (AA) is 27,730 and the number of sequences assigned to IMGT clonotypes (AA) is 36,759 (36,722 “one copy” + 37 “more than one” = 36,759) (Figure 1). For S2, the number of IMGT clonotypes (AA) is 17,302 and the number of sequences assigned to IMGT clonotypes (AA) is 23,815 (23,800 “one copy” + 15 “more than one” = 23,815).

Unselected IMGT clonotypes (AA) that correspond to CDR3-IMGT length outliers are listed separately. Seven IMGT clonotypes (AA) with an outlier CDR3-IMGT length (<4 AA or >45 AA) were removed: one from S1 (Figure 1) and six from S2.

### 3.2 Statistical Test Results

“Statistical test results” (Figure 2) displays the results obtained by applying the standardized procedure described in Ref. (9) (Table S1 in Supplementary Material) (13–19) to evaluate the significance of pairwise comparisons between differences in proportions of the IMGT clonotype (AA) diversity or expression, per V, D, or J gene of a given IG or TR group, from IMGT/HighV-QUEST statistical output.

As an improvement of the procedure, results are provided not only for IMGT genes as described in Ref. (9) but also for IMGT alleles. In the case of individuals heterozygous for a given gene, it becomes possible to detect if significant differences in gene proportions, validated by all multiple testing procedures, depend on one allele or not.

In the left panel, users can choose between the display of IMGT clonotype (AA) diversity or expression results and select the columns to show.

“Statistical test results” displays results for genes and alleles in tables of 21 columns (content described in Table S2 in Supplementary Material). Column filters are provided for each table to select the gene name (column “Gene_Name”), to search a specific gene type (e.g., typing V, D, or J in column “Gene_Type”) or a specific test result (e.g., typing “rawp” in the last column “Test_interpretation” to show significant differences in proportions before adjustment of *p*-values). For numeric values, sliders are displayed in column filters to delimit a value range (e.g., in column “rawp,” when the slider of unadjusted *p*-values range is fixed from 0 to 0.05, significant differences in proportions before adjustment are shown). Results can also be ordered based on a specific column by clicking on its label (double click to switch from ascending to descending order).

In the case of null or small gene (resp., allele) occurrences when

$$n_{1}p_{1}^{k}<5,n_{1}(1-p_{1}^{k})<5\text{ and }n_{2}p_{2}^{k}<5,n_{2}(1-p_{2}^{k})<5$$

where *n*_1_ (resp., *n*_2_), number of IMGT clonotypes (AA) in set 1 (resp., set 2); *k*, IMGT gene name; and $p_{1}^{k}$ (resp., $p_{2}^{k}$), proportion of the gene *k* in set 1 (resp., set 2), the *z*-score is not applicable and results displayed in column “*z*” are not considered. Fisher’s exact test is used instead and associated *p*-values are displayed in column “rawp” of the “Statistical test results for genes” (resp., “Statistical test results for alleles”) table (Figure 2). Multiple testing procedures are applied for *p*-values returned by the Fisher’s exact test.

The list of genes with null or small occurrences is listed below the table. The same information is given for analyzed alleles of genes having significant differences in proportions validated by all multiple testing procedures.

It is possible to modify the display for several or single genes or alleles, as defined in Ref. (5), without modifying the results. Thus, for genes, by default, the display shows “Several genes,” clicking on “Single genes” radio button allows to hide them. For alleles, by default, the display shows “Single alleles,” clicking on “Several alleles” radio button allows to show them.

Taking the example of “S1” and “S2,” for genes, 72 hypotheses are tested, corresponding to 41 genes for the IGHV group, 25 genes for the IGHD group and 6 genes for the IGHJ group. The sets are assumed as independent and individual tests are independent of each others (9). For alleles, 55 hypotheses are tested, corresponding to 34 alleles for the IGHV group, 12 alleles for the IGHD group, and 9 alleles for the IGHJ group.

### 3.3 Multiple Testing Procedures Plots

“Multiple testing procedures plots” (Figure 3) displays multiple testing procedures visualization plots as line graphs (left figures) and scatter plots (right figures), for genes and alleles, for the comparison of the differences in proportions for IMGT clonotypes (AA) with a gene of a given group (IGHV, IGHD, IGHJ), between sets 1 and 2.

The line graphs allow (by hovering with the mouse) the visualization of the exact number of rejected null hypotheses (number of significant differences in proportions) for a chosen α-level (Type I error rate) under a given procedure (Bonferroni, Holm, Hochberg, ŠidákSS, and ŠidákSD) (9). For “S1” and “S2” genes, the line graph shows that 47 differences in proportions are significant before the adjustment of *p*-values (black line), whereas from 32 to 46 are validated after adjustment by all multiple testing procedures. For “S1” and “S2” alleles, the line graph shows 44 before adjustment and from 37 to 43 after adjustment, respectively, and highlights that the BH procedure should be chosen to keep as many significant differences as possible. These graphs also allow the identification, for a selected number of rejected null hypotheses, of the α-level that is required to get that number for a given procedure (9).

The scatter plots complete the information given by the line graphs by specifying the sign of the difference in proportions. They show negative decimal logarithms (−log_10_) of unadjusted *p*-values (black symbols) and adjusted *p*-values obtained by each multiple testing procedure (colored symbols) against test statistics (*z*-scores). Hovering with the mouse over the scatter plot points allows to see the gene name with the coordinates (x: *z*-score, y: −log(*p*-values,10)). Corresponding values of the scatter plots are reported in a table below the figures and highlighted values in yellow correspond to significant differences in proportions.

For “S1” and “S2” genes, the 47 significant differences in proportions before the adjustment of *p*-values cited above include 32 negative differences and 15 positive differences. For “S1” and “S2” alleles, the 44 differences in proportions before adjustment include 24 negative differences and 20 positive differences. These values can be found using the slider in “*z* (test statistic)” column filter of the table, by displaying only *z*-score values less than 1.96 (for significant negative differences) or *z*-score values greater than 1.96 (for significant positive differences).

### 3.4 Synthesis Graph

“Synthesis graph” (Figure 4) displays the synthesis graph that combines a normalized bar graph of gene proportions and the differences in proportions with significance and confidence intervals (CI) (9). The same information is given for alleles of genes having significant differences in proportions validated by all multiple testing procedures.

The normalized bar graph represents the numbers of IMGT clonotypes (AA) for a given gene obtained from the IMGT/HighV-QUEST outputs normalized for 10,000 IMGT clonotypes (AA) (for clonotype diversity) or for 10,000 sequences assigned to IMGT clonotypes (AA) (for clonotype expression).

In the left panel, users can select IMGT clonotype diversity or expression and the gene type (V, D, or J).

“Synthesis graph” for genes permits a visual comparative analysis of IMGT clonotype (AA) diversity or expression for each gene per group (V, D, or J) between sets 1 and 2 and facilitates the comparison with the experimental result.

Displayed IMGT gene names are ordered by their positions in the locus with all known functionalities according to the IMGT Scientific chart (4) and to IMGT/GENE-DB (20). In the example, in Figure 4, the most important differences in proportions of IMGT clonotypes (AA) of the IGHV4-34 and IGHV3-23 genes with higher IMGT clonotype (AA) diversity in “S1” (IgD^+^) compared to “S2” (IgD^–^) are validated by all multiple testing procedures at an α-level = 0.05. The highest clonotype diversity in S2 (IgD^−^) compared to S1 (IgD^+^) is represented by the gene IGHV1-8 (*P* < 10^−6^, indicated in “Statistical test results”). “Synthesis graph” for alleles displayed IMGT allele names ordered in ascending order. In Figure 4, the synthesis graph shows that this highest IMGT clonotype (AA) diversity of the genes mentioned above is represented mainly by the alleles IGHV4-34*01 (S1), IGHV3-23*04 (S1), and IGHV1-8*01 (S2).

This type of analysis can be used for the characterization of haplotypes in individuals in relation with their expressed repertoire and for an evaluation of the sequences of “alleles” poorly represented or unexpected, which may result of too short or mutated sequences.

### 3.5 CDR-IMGT Lengths

“CDR-IMGT lengths” (Figure 5) displays interactive bar graphs for sets 1 and 2 showing the distribution of the number of IMGT clonotypes (AA) (for IMGT clonotype diversity) or of the number of sequences assigned to IMGT clonotypes (AA) (for IMGT clonotype expression) per CDR-IMGT length for a given CDR-IMGT type (1, 2 or 3) (CDR-IMGT length and numbers between parentheses are visible hovering with the mouse). The distribution of the lengths of the CDR1-IMGT and CDR2-IMGT (encoded by the V gene) depends on the usage of the different V genes and subgroups, whereas the distribution of the lengths of the CDR3-IMGT (which result from the V-(D)-J rearrangement) characterizes the junction of the IMGT clonotypes. CDR-IMGT lengths and positions are based on the IMGT unique numbering for V domains (21).

In the left panel, users can select IMGT clonotype diversity or expression and the CDR-IMGT type (1, 2 or 3). “CDR-IMGT lengths” displays, in Figure 5, the distribution per CDR3-IMGT length. The interactive graph displays, for each set, the CDR-IMGT length with, between parentheses, the absolute number of IMGT clonotypes (AA) or the absolute number of sequences assigned to IMGT clonotypes (AA). The bar graphs for sets 1 and 2 look very similar with a peak of CDR3-IMGT having 15 AA. There is the possibility to zoom in by clicking and dragging over the graph area if users are interested by a specific range of CDR-IMGT lengths (double clicking again to zoom out). Those details are in the documentation of the tool.^3^

On a practical basis, bar graphs for CDR3-IMGT can be a useful visualization to detect the presence of some lengths that should be considered as outliers (few clonotypes with very high or very low CDR3-IMGT length in one or both of compared sets) and users can remove them from the analysis by modifying the CDR3-IMGT length range in the left panel.

By clicking on “Normalized values” button in the left panel, “CDR-IMGT lengths” are displayed with normalized values (i.e., numbers of IMGT clonotypes (AA) for a given gene normalized for 10,000 IMGT clonotypes (AA) (for clonotype diversity) or for 10,000 sequences assigned to IMGT clonotypes (AA) (for clonotype expression)).

The users have also the possibility to list the CDR3-IMGT sequences of the IMGT clonotypes (AA) for a given CDR3-IMGT length in sets 1 and 2 (by choosing a length in the left panel). The lists are displayed below the bar graphs. For example, the lists of the CDR3-IMGT sequences of the IMGT clonotypes (AA) for the selected length of 15 AA is shown in Figure 5 for sets 1 and 2.

### 3.6 CDR-IMGT AA Properties

“CDR-IMGT AA properties” (Figure 6) displays the distribution of the IMGT classes of the 20 common amino acids (22) at CDR-IMGT positions (21) in sets 1 and 2. IMGT AA classes include “Physicochemical,” “Hydropathy,” “Volume,” “Chemical,” “Charge,” “Hydrogen donor or acceptor atoms,” and “Polarity” (detailed in Table S3 in Supplementary Material). Comparisons of two sets are useful to detect characteristics of amino acids at positions important for the V domain antibody diversity or, by contrast, for maintaining its structure. As mentioned above, CDR1-IMGT and CDR2-IMGT depend on the V gene usage, whereas CDR3-IMGT characterizes the IMGT clonotype junction.

In the left panel, users select the CDR-IMGT type (1, 2, or 3), then the CDR-IMGT length to be displayed and the IMGT classes of the 20 amino acids for sets 1 and 2.

“CDR-IMGT AA properties” displays by default stacked bar graphs showing the distribution of the 20 amino acids at a specific CDR-IMGT position with a table reporting the frequencies of the 20 amino acids (in rows) at CDR-IMGT positions (in columns). Interactive stacked bar graphs are generated for each set in order to visualize frequencies at a specific CDR-IMGT position for a selected IMGT AA class.

To measure and visualize position diversity, variability plots based on “Shannon entropy” (23), “Wu-Kabat variability” (24), or “Simpson index” (25) indexes are displayed, for sets 1 and 2, with corresponding summary tables.

This feature is also provided by the tool presented in Ref. (26) but, with IMGT/StatClonotype, results can be obtained for the different IMGT AA classes (22) as indicated above and colored according to the IMGT Color menu for each class given in Table S3 in Supplementary Material.

Both, Shannon and Simpson indexes measure the variability of different AA (or IMGT AA class representatives) at a given CDR-IMGT position, whereas Wu-Kabat index is the ratio between the number of different AA (or IMGT AA class representatives) at a given position and the frequency of the most represented AA (or IMGT AA class representatives) at that position (24). Positions with Shannon entropy values greater than 2 are generally considered as variable and those less than 1 are considered as highly conserved (27). For Simpson diversity index, values range from 0 and 1, the greater the value, the greater the position diversity. For Wu–Kabat variability coefficient, higher values indicate higher position diversity.

In Figure 6, the variability plot for “S1” and “S2” shows that the VH CDR3-IMGT (length: 13AA) positions 105 and 106 (which correspond to the 3′V-REGION) and 115, 116, and 117 (which correspond to the 5′J-REGION) are considered as conserved (Shannon entropy values from 0.217 to 1.882 for “S1” and from 0.465 and 1.927 for “S2” (tables at the bottom of Figure 6)). By contrast, positions 107 to 114, as expected from the IGH V-D-J mechanism of rearrangement (1), show a greater diversity (Shannon entropy values from 2.459 to 2.733 for “S1” and from 2.497 to 2.731 for “S2”).

### 3.7 V–D–J Gene Associations

“V–D–J gene associations” (Figure 7) displays interactive heatmaps to represent V–J, V–D, or D–J gene associations in sets 1 and 2. In the left panel, users select V–J, V–D, or D–J gene association for sets 1 and 2. If the option “Results with clustering” is unchecked, heatmaps are shown without dendrograms and ordering. Heatmaps, in this case, are a visualization of contingency tables crossing different gene types (V–J, V–D, or D–J) associations.

Results can be given in normalized values. If the option “Results with clustering” is checked, a double Ward hierarchical clustering with Euclidean distance is performed (as usually required for software that implements Ward’s method, the algorithm checks whether the function arguments specify Euclidean distances). This type of classification operates simultaneously on the lines and columns of a matrix intersecting two different types of genes.

“V-D-J gene associations” displays the heatmaps (clustered or not) for sets 1 and 2 with corresponding summary tables. Tables crossing the V–J, V–D, or D–J gene occurrences in sets 1 and 2 are given below heatmaps. In Figure 7, the V–J gene associations for sets 1 and 2 with the option “Results with clustering” are shown. The rows (columns) of the central card of the heatmap are ordered such that similar rows (columns) are close to each other. On vertical and horizontal margins are represented dendrograms that gave rise to these classifications. The dendrograms in the horizontal margin of the two heatmaps highlight three groupings of the IGHJ genes:{IGHJ4}, {IGHJ1, IGHJ2}, and {IGHJ3, IGHJ5, IGHJ6}. Darker colors in heatmaps indicate mainly the strong associations of the IGHJ4 gene with IGHV4-34 and IGHV3-23 genes for “S1” and with IGHV4-34, IGHV4-31, IGHV1-2, IGHV4-61, IGHV4-39, IGHV4-59, IGHV1-8, IGHV3-3, and IGHV3-23 genes for “S2.”

This clustering is picking up on overall response rather than pattern because we do not fix pattern to be studied. The use of the Euclidean metrics regroups genes having the closest distances based on the occurrences in each set. Such an analysis permits to detect genes with similar diversity or expression profiles which can be further explored for given and/or related specificities in immune repertoire comparative analysis.

## 4 Availability and Future Directions

The package “IMGTStatClonotype” is freely available as a downloadable standalone package under the LGPL license at IMGT^®^, see text footnote 1, with full documentation and a convivial interface on the user’s web browser. “IMGTStatClonotype” is installable under Windows, Linux, and Mac OSx operating systems. All R package dependencies (reshape2 (28), data.table (29), ggplot2 (30), gridExtra (31), DT (32), shiny (11), shinyjs (33), plotly (34), and d3heatmap (35)) are available from Comprehensive R Archive Network (CRAN) except for the package multtest (36) available from Bioconductor only. For each new feature added to IMGT/HighV-QUEST, our intent is to advance the development of IMGT/StatClonotype tool in terms of analysis processing and visualization.

Currently, IMGT/StatClonotype allows users to perform standardized pairwise comparison of NGS IG or TR data from IMGT/HighV-QUEST statistical output. Based on properties of multiple testing procedures (9) (Table S1 in Supplementary Material), there is no definitive choice on the multiple testing procedure that should be used. Generally, users can choose familiar procedure with their audience or their field of study. Besides, there is maybe some logic to make a choice. For example, in a preliminary study, users have interest to choose less conservative procedures (e.g., BH and BY procedures) to keep as many significant differences as possible and not exclude interesting gene variations in future studies. By contrast, in medical studies where people’s lives are involved and costly vaccines or drugs are contemplated, users should opt for procedures of very high level of certainty (i.e., significant differences in proportions validated by all multiple testing procedures or the most conservative) before concluding that vaccine or drug is better than another. It was a decision not to restrict the analysis to one given procedure. For that reason, the strong point of IMGT/StatClonotype is to allow users to make the appropriate choice easily as long as results given by different multiple testing procedures are shown at the same time.

IMGT/StatClonotype, based on the standardized IMGT/HighV-QUEST output, provides a generic statistical procedure and integrated features for the comparative analysis of CDR-IMGT and V–D–J associations.

By these functionalities, IMGT/StatClonotype is suitable for detecting significant changes in IG and TR immunoprofiles in protective (vaccination, cancers, and infections) or pathogenic (autoimmunity and lymphoproliferative disorders) immune responses.

## Author Contributions

SA and M-PL conceived and designed the experiments. SA designed the algorithm and implemented the tool. SA and M-PL wrote the paper. PD, DM, VG, SK, and M-PL supervised the project. All the authors have read and approved the final manuscript.

## Conflict of Interest Statement

The authors declare that the research was conducted in the absence of any commercial or financial relationships that could be construed as a potential conflict of interest.

## Footnotes

^2^http://www.imgt.org

^3^http://www.imgt.org/StatClonotype/IMGTStatClonotypeDoc.html
