# Supplementary material for: IMGT/StatClonotype for Pairwise Evaluation and Visualization of NGS IG and TR IMGT Clonotype (AA) Diversity or Expression from IMGT/HighV-QUEST
Source: Front Immunol. 2016 Sep 9;7:339. doi: 10.3389/fimmu.2016.00339 (PMC5016520; doi:10.3389/fimmu.2016.00339)
Supplement: Supplementary file 1 [file Data_Sheet_1.pdf]

Supplementary Material: IMGT/StatClonotype for pairwise evaluation and visualization of NGS IG and TR IMGT clonotype (AA) diversity or expression from IMGT/HighV-QUEST

Safa Aouinti[1,2,*], Véronique Giudicelli[1], Patrice Duroux[1], Dhafer Malouche[2], Sofia Kossida[1,*], Marie-Paule Lefranc[1,*]

*Correspondence:
Safa Aouinti, Sofia Kossida, Marie-Paule Lefranc
safa.aouinti@igh.cnrs.fr, sofia.kossida@igh.cnrs.fr, marie-paule.lefranc@igh.cnrs.fr

1 SUPPLEMENTARY TABLES

Table S1. Properties of the multiple testing procedures. These procedures are used in IMGT/StatClonotype for multiple testing error rate control (9).

**Procedures**	**Type of control**	**Algorithm structure**	**Dependence of *p*-values under** H_0	**Properties**
**Bonferonni** [13]	FWER	*Single-step*	Ignorance	The most conservative
**Šidák (SS)** [14,15]	FWER	*Single-step*	Independence	Less conservative than Bonferroni
**Holm** [16]	FWER	*Step-down*	Ignorance	Less conservative than Bonferroni
**Šidák (SD)** [14.15]	FWER	*Step-down*	Dependence	Similar to Holm
**Hochberg** [17]	FWER	*Step-up*	Independence	*Step-up* of Holm
**Benjamini & Hochberg (BH)** [18]	FDR	*Step-up*	Independence	The least conservative
**Benjamini & Yekutieli (BY)** [19]	FDR	*Step-up*	Ignorance	More conservative than BH

- FWER (Family-wise error rate): the probability to make one or more false positives.
- FDR (False discovery rate): the expected proportion of false positives.

Table S2. Statistical test results table headers. Headers definitions correspond to their use for significance results using multiple testing procedures (9).

Table column	Description
**Gene_Name**	The list of IMGT gene names found in the two compared sets from the IMGT/HighV-QUEST output
**Gene_Type**	The type of genes (V, D or J)
**Nb_IMGT_clonotype_AA.set1**	The nb of IMGT clonotypes (AA) in the first set from the IMGT/HighV-QUEST output with the corresponding gene indicated in the first column 'Gene_Name'
**Proportion.set1**	The proportion of IMGT clonotypes (AA) in the first set from the IMGT/HighV-QUEST output with the corresponding gene indicated in the first column 'Gene_Name'
**Normalized_proportion.set1**	The normalized proportion for 10,000 IMGT clonotypes (AA) in the first IMGT/HighV-QUEST output (set) with the corresponding gene indicated in the first column 'Gene_Name'
**Nb_IMGT_clonotype_AA.set2**	The nb of IMGT clonotypes (AA) in the second set from the IMGT/HighV-QUEST output with the corresponding gene indicated in the first column 'Gene_Name'
**Proportion.set2**	The proportion of IMGT clonotypes (AA) in the second set from the IMGT/HighV-QUEST output with the corresponding gene indicated in the first column 'Gene_Name'
**Normalized_proportion.set2**	The normalized proportion for 10,000 IMGT clonotypes (AA) in the second IMGT/HighV-QUEST output (set) with the corresponding gene indicated in the first column 'Gene_Name'
**Difference_proportion**	The difference in proportions of IMGT clonotypes (AA) in the two compared sets from the IMGT/HighV-QUEST output with the corresponding gene indicated in the first column 'Gene_Name'
**z**	The z-score values to determine the significance of the difference between two proportions
**Lower_bound_IC_diff_prop**	The lower bound of the 95% confidence interval (CI) for the difference in proportions of IMGT clonotypes (AA) in the two compared sets from the IMGT/HighV-QUEST output
**Upper_bound_IC_diff_prop**	The upper bound of the 95% confidence interval (CI) for the difference in proportions of IMGT clonotypes (AA) in the two compared sets from the IMGT/HighV-QUEST output
**rawp**	The p-values obtained from z-scores to evaluate the significance of difference in proportions of IMGT clonotypes (AA) in the two compared sets from the IMGT/HighV-QUEST output
**Bonferroni**	The adjusted p-values issued from the Bonferroni multiple testing procedure
**Holm**	The adjusted p-values issued from the Holm multiple testing procedure
**Hochberg**	The adjusted p-values issued from the Hochberg multiple testing procedure
**ŠidákSS**	The adjusted p-values issued from the Šidák single-step (SS) multiple testing procedure
**ŠidákSD**	The adjusted p-values issued from the Šidák single-down (SD) multiple testing procedure
**BH**	The adjusted p-values issued from the Benjamini & Hochberg (BH) multiple testing procedure
**BY**	The adjusted p-values issued from the Benjamini & Yekutieli (BY) multiple testing procedure
**Test_interpretation**	The test interpretation: before adjustment of p-values (rawp) non-significant and after adjustment by the multiple testing procedure: significant differences in proportions validated by the seven procedures (All_p), by two or more procedures (Min_2p) and only by BH (Only_BH)

Table S3. IMGT classes of the 20 common amino acids side chain properties
http://www.imgt.org/IMGTeducation/Aide-memoire/_UK/aminoacids/IMGTclasses.html
(from IMGT®, the international ImMunoGeneTics information system® (3), http://www.imgt.org).

Amino acid	Abbreviations		IMGT classes of the amino acids side chain properties [15]						
			Hydropathy (3 classes)	Volume (5 classes)	Chemical (7 classes)	Physicochemical (11 classes)	Charge (3 classes)	Polarity (2 classes)	Hydrogen donor or acceptor atom (4 classes)
Alanine	Ala	A	hydrophobic (1)	very small	aliphatic (1)	aliphatic (1)	uncharged(3)	nonpolar (2)	none (4)
Arginine	Arg	R	hydrophilic (3)	large (4)	basic (5)	basic (5)	positive charged (1)	polar (1)	donor (1)
Asparagine	Asn	N	hydrophilic (3)	small (2)	amide (7)	amide (2)	uncharged (3)	polar (1)	donor and acceptor (3)
Aspartic acid	Asp	D	hydrophilic (3)	small (2)	acidic (6)	acidic (6)	negative charged (2)	polar (1)	acceptor (2)
Cysteine	Cys	C	hydrophobic (1)	small (2)	sulfur (3)	sulfur (3)	uncharged (3)	nonpolar (2)	none (4)
Glutamine	Gln	Q	hydrophilic (3)	medium (3)	amide (7)	amide (2)	uncharged (3)	polar (1)	donor and acceptor (3)
Glutamic acid	Glu	E	hydrophilic (3)	medium (3)	acidic (6)	acidic (6)	negative charged (2)	polar (1)	acceptor (2)
Glycine	Gly	G	neutral (2)	very small	aliphatic (1)	G (11)	uncharged (3)	nonpolar (2)	none (4)
Histidine	His	H	neutral (2)	medium (3)	basic (5)	basic (5)	positive charged (1)	polar (1)	donor and acceptor (3)
Isoleucine	Ile	I	hydrophobic (1)	large (4)	aliphatic (1)	aliphatic (1)	uncharged (3)	nonpolar (2)	none (4)
Leucine	Leu	L	hydrophobic (1)	large (4)	aliphatic (1)	aliphatic (1)	uncharged (3)	nonpolar (2)	none (4)
Lysine	Lys	K	hydrophilic (3)	large (4)	basic (5)	basic (5)	positive charged (1)	polar (1)	donor (1)
Methionine	Met	M	hydrophobic (1)	large (4)	sulfur (3)	sulfur (3)	uncharged (3)	nonpolar (1)	none (4)
Phenylalanine	Phe	F	hydrophobic (1)	very large (5)	aromatic (2)	F (7)	uncharged (3)	nonpolar (1)	none (4)
Proline	Pro	P	neutral (2)	small (2)	aliphatic (1)	P (10)	uncharged (3)	nonpolar (1)	none (4)
Serine	Ser	S	neutral (2)	very small	hydroxyl (4)	hydroxyl (4)	uncharged (3)	polar (2)	donor and acceptor (3)
Threonine	Thr	T	neutral (2)	small (2)	hydroxyl (4)	hydroxyl (4)	uncharged (3)	polar (2)	donor and acceptor (3)
Tryptophan	Trp	W	hydrophobic (1)	very large (5)	aromatic (2)	W (8)	uncharged (3)	nonpolar (1)	donor (1)
Tyrosine	Tyr	Y	neutral (2)	very large (5)	aromatic (2)	Y (9)	uncharged (3)	polar (2)	donor and acceptor (3)
Valine	Val	V	hydrophobic (1)	medium (3)	aliphatic (1)	aliphatic (1)	uncharged (3)	nonpolar (1)	none (4)

- Numbers between parentheses indicate the class number, for a given property.
- Color codes of each class (1): `http://www.imgt.org/IMGTScientificChart/RepresentationRules/colormenu.php`
